# National Estimates of Gender-Affirming Surgery in the US

**DOI:** 10.1001/jamanetworkopen.2023.30348

**Published:** 2023-08-23

**Authors:** Jason D. Wright, Ling Chen, Yukio Suzuki, Koji Matsuo, Dawn L. Hershman

**Affiliations:** 1 Department of Obstetrics and Gynecology, Columbia University College of Physicians and Surgeons, New York, New York; 2 Department of Obstetrics and Gynecology, University of Southern California, Los Angeles

## Abstract

**Importance:**

While changes in federal and state laws mandating coverage of gender-affirming surgery (GAS) may have led to an increase in the number of annual cases, comprehensive data describing trends in both inpatient and outpatient procedures are limited.

**Objective:**

To examine trends in inpatient and outpatient GAS procedures in the US and to explore the temporal trends in the types of GAS performed across age groups.

**Design, Setting, and Participants:**

This cohort study includes data from 2016 to 2020 in the Nationwide Ambulatory Surgery Sample and the National Inpatient Sample. Patients with diagnosis codes for gender identity disorder, transsexualism, or a personal history of sex reassignment were identified, and the performance of GAS, including breast and chest procedures, genital reconstructive procedures, and other facial and cosmetic surgical procedures, were identified.

**Main Outcome Measures:**

Weighted estimates of the annual number of inpatient and outpatient procedures performed and the distribution of each class of procedure overall and by age were analyzed.

**Results:**

A total of 48 019 patients who underwent GAS were identified, including 25 099 (52.3%) who were aged 19 to 30 years. The most common procedures were breast and chest procedures, which occurred in 27 187 patients (56.6%), followed by genital reconstruction (16 872 [35.1%]) and other facial and cosmetic procedures (6669 [13.9%]). The absolute number of GAS procedures rose from 4552 in 2016 to a peak of 13 011 in 2019 and then declined slightly to 12 818 in 2020. Overall, 25 099 patients (52.3%) were aged 19 to 30 years, 10 476 (21.8%) were aged 31 to 40, and 3678 (7.7%) were aged12 to 18 years. When stratified by the type of procedure performed, breast and chest procedures made up a greater percentage of the surgical interventions in younger patients, while genital surgical procedures were greater in older patients.

**Conclusions and Relevance:**

Performance of GAS has increased substantially in the US. Breast and chest surgery was the most common group of procedures performed. The number of genital surgical procedures performed increased with increasing age.

## Key Points

**Question:**

What are the temporal trends in gender-affirming surgery (GAS) in the US?

**Findings:**

In this cohort study of 48 019 patients, GAS increased significantly, nearly tripling from 2016 to 2019. Breast and chest surgery was the most common class of procedures performed overall; genital reconstructive procedures were more common among older individuals.

**Meaning:**

These findings suggest that there will be a greater need for clinicians knowledgeable in the care of transgender individuals with the requisite expertise to perform gender-affirming procedures.

## Teaser

This cohort study examines trends in inpatient and outpatient gender-affirming surgical procedures in the US and explores the temporal trends in the types of procedures across age groups.

## Introduction

Gender dysphoria is characterized as an incongruence between an individual’s experienced or expressed gender and the gender that was assigned at birth.^1^ Transgender individuals may pursue multiple treatments, including behavioral therapy, hormonal therapy, and gender-affirming surgery (GAS).^2^ GAS encompasses a variety of procedures that align an individual patient’s gender identity with their physical appearance.^2,3,4^

While numerous surgical interventions can be considered GAS, the procedures have been broadly classified as breast and chest surgical procedures, facial and cosmetic interventions, and genital reconstructive surgery.^2,4^ Prior studies^2,3,4,5,6,7^ have shown that GAS is associated with improved quality of life, high rates of satisfaction, and a reduction in gender dysphoria. Furthermore, some studies have reported that GAS is associated with decreased depression and anxiety.^8^ Lastly, the procedures appear to be associated with acceptable morbidity and reasonable rates of perioperative complications.^2,4^

Given the benefits of GAS, the performance of GAS in the US has increased over time.^9^ The increase in GAS is likely due in part to federal and state laws requiring coverage of transition-related care, although actual insurance coverage of specific procedures is variable.^10,11^ While prior work has shown that the use of inpatient GAS has increased, national estimates of inpatient and outpatient GAS are lacking.^9^ This is important as many GAS procedures occur in ambulatory settings. We performed a population-based analysis to examine trends in GAS in the US and explored the temporal trends in the types of GAS performed across age groups.

## Methods

### Data Sources

To capture both inpatient and outpatient surgical procedures, we used data from the Nationwide Ambulatory Surgery Sample (NASS) and the National Inpatient Sample (NIS). NASS is an ambulatory surgery database and captures major ambulatory surgical procedures at nearly 2800 hospital-owned facilities from up to 35 states, approximating a 63% to 67% stratified sample of hospital-owned facilities. NIS comprehensively captures approximately 20% of inpatient hospital encounters from all community hospitals across 48 states participating in the Healthcare Cost and Utilization Project (HCUP), covering more than 97% of the US population. Both NIS and NASS contain weights that can be used to produce US population estimates.^12,13^ Informed consent was waived because data sources contain deidentified data, and the study was deemed exempt by the Columbia University institutional review board. This cohort study followed the Strengthening the Reporting of Observational Studies in Epidemiology (STROBE) reporting guideline.

### Patients and Procedures

We selected patients of all ages with an *International Statistical Classification of Diseases and Related Health Problems, Tenth Revision *(*ICD-10*) diagnosis codes for gender identity disorder or transsexualism (*ICD-10* F64) or a personal history of sex reassignment (*ICD-10* Z87.890) from 2016 to 2020 (eTable in Supplement 1). We first examined all hospital (NIS) and ambulatory surgical (NASS) encounters for patients with these codes and then analyzed encounters for GAS within this cohort. GAS was identified using *ICD-10* procedure codes and *Common Procedural Terminology* codes and classified as breast and chest procedures, genital reconstructive procedures, and other facial and cosmetic surgical procedures.^2,4^ Breast and chest surgical procedures encompassed breast reconstruction, mammoplasty and mastopexy, or nipple reconstruction. Genital reconstructive procedures included any surgical intervention of the male or female genital tract. Other facial and cosmetic procedures included cosmetic facial procedures and other cosmetic procedures including hair removal or transplantation, liposuction, and collagen injections (eTable in Supplement 1). Patients might have undergone procedures from multiple different surgical groups. We measured the total number of procedures and the distribution of procedures within each procedural group.

Within the data sets, sex was based on patient self-report. The sex of patients in NIS who underwent inpatient surgery was classified as either male, female, missing, or inconsistent. The inconsistent classification denoted patients who underwent a procedure that was not consistent with the sex recorded on their medical record. Similar to prior analyses, patients in NIS with a sex variable not compatible with the procedure performed were classified as having undergone genital reconstructive surgery (GAS not otherwise specified).^9^

### Covariates

Clinical variables in the analysis included patient clinical and demographic factors and hospital characteristics. Demographic characteristics included age at the time of surgery (12 to 18 years, 19 to 30 years, 31 to 40 years, 41 to 50 years, 51 to 60 years, 61 to 70 years, and older than 70 years), year of the procedure (2016-2020), and primary insurance coverage (private, Medicare, Medicaid, self-pay, and other). Race and ethnicity were only reported in NIS and were classified as White, Black, Hispanic and other. Race and ethnicity were considered in this study because prior studies have shown an association between race and GAS. The income status captured national quartiles of median household income based of a patient’s zip code and was recorded as less than 25% (low), 26% to 50% (medium-low), 51% to 75% (medium-high), and 76% or more (high). The Elixhauser Comorbidity Index was estimated for each patient based on the codes for common medical comorbidities and weighted for a final score.^14^ Patients were classified as 0, 1, 2, or 3 or more. We separately reported coding for HIV and AIDS; substance abuse, including alcohol and drug abuse; and recorded mental health diagnoses, including depression and psychoses. Hospital characteristics included a composite of teaching status and location (rural, urban teaching, and urban nonteaching) and hospital region (Northeast, Midwest, South, and West). Hospital bed sizes were classified as small, medium, and large. The cutoffs were less than 100 (small), 100 to 299 (medium), and 300 or more (large) short-term acute care beds of the facilities from NASS and were varied based on region, urban-rural designation, and teaching status of the hospital from NIS.^8^ Patients with missing data were classified as the unknown group and were included in the analysis.

### Statistical Analysis

National estimates of the number of GAS procedures among all hospital encounters for patients with gender identity disorder were derived using discharge or encounter weight provided by the databases.^15^ The clinical and demographic characteristics of the patients undergoing GAS were reported descriptively. The number of encounters for gender identity disorder, the percentage of GAS procedures among those encounters, and the absolute number of each procedure performed over time were estimated. The difference by age group was examined and tested using Rao-Scott χ^2^ test. All hypothesis tests were 2-sided, and *P* < .05 was considered statistically significant. All analyses were conducted using SAS version 9.4 (SAS Institute Inc).

## Results

A total of 48 019 patients who underwent GAS were identified (Table 1). Overall, 25 099 patients (52.3%) were aged 19 to 30 years, 10 476 (21.8%) were aged 31 to 40, and 3678 (7.7%) were aged 12 to 18 years. Private insurance coverage was most common in 29 064 patients (60.5%), while 12 127 (25.3%) were Medicaid recipients. Depression was reported in 7192 patients (15.0%). Most patients (42 467 [88.4%]) were treated at urban, teaching hospitals, and there was a disproportionate number of patients in the West (22 037 [45.9%]) and Northeast (12 396 [25.8%]). Within the cohort, 31 668 patients (65.9%) underwent 1 procedure while 13 415 (27.9%) underwent 2 procedures, and the remainder underwent multiple procedures concurrently (Table 1).

**Table 1.** Demographics of Transgender Patients Undergoing Gender-Affirming Surgery Overall and Stratified by Classes of Gender-Affirming Surgery

Characteristic	Overall		Breast/chest surgery		Genital surgery		Other cosmetic procedures	
	No. (SE)	% (SE)	No. (SE)	% (SE)	No. (SE)	% (SE)	No. (SE)	% (SE)
Age, y								
12-18	3678 (272)	7.7 (0.3)	3215 (258)	11.8 (0.5)	405 (54)	2.4 (0.3)	350 (53)	5.3 (0.7)
19-30	25 099 (1442)	52.3 (0.6)	16 067 (1166)	59.1 (0.6)	7461 (437)	44.2 (0.8)	2946 (246)	44.2 (1.2)
31-40	10 476 (646)	21.8 (0.4)	4918 (384)	18.1 (0.4)	4423 (309)	26.2 (0.6)	1729 (165)	25.9 (1.0)
41-50	4359 (266)	9.1 (0.3)	1650 (132)	6.1 (0.3)	2168 (155)	12.8 (0.5)	784 (77)	11.8 (0.6)
51-60	2958 (193)	6.2 (0.2)	949 (78)	3.5 (0.2)	1546 (124)	9.2 (0.5)	610 (69)	9.1 (0.7)
61-70	1271 (92)	2.6 (0.2)	350 (33)	1.3 (0.1)	742 (68)	4.4 (0.3)	229 (31)	3.4 (0.4)
>70	177 (26)	0.4 (0.1)	37 (8)	0.1 (0)	126 (23)	0.7 (0.1)	19 (6)	0.3 (0.1)
Unknown	3 (2)	0	0	0	1 (1)	0	2 (2)	0
Sex								
Male	15 234 (965)	31.7 (0.8)	8707 (639)	32.0 (0.7)	5417 (460)	32.1 (1.7)	2144 (180)	32.1 (1.3)
Female	26 264 (1584)	54.7 (1.0)	17 852 (1294)	65.7 (0.5)	5455 (315)	32.3 (1.6)	4419 (386)	66.3 (1.3)
Unknown	6522 (612)	13.6 (1.1)	627 (137)	2.3 (0.5)	6000 (585)	35.6 (2.2)	106 (20)	1.6 (0.3)
Race, inpatient^a^								
White	6915 (642)	65.1 (2.0)	575 (77)	58.4 (4.2)	6050 (595)	67.8 (2.0)	635 (155)	53.1 (6.2)
Black	955 (123)	9.0 (1.0)	125 (28)	12.7 (2.5)	720 (105)	8.1 (1.0)	145 (36)	12.1 (3.1)
Hispanic	1050 (130)	9.9 (0.9)	130 (31)	13.2 (2.6)	820 (117)	9.2 (0.9)	140 (38)	11.7 (3.0)
Other	1380 (253)	13.0 (1.9)	95 (24)	9.6 (2.1)	1060 (188)	11.9 (1.7)	255 (82)	21.3 (5.0)
Unknown	325 (64)	3.1 (0.6)	60 (24)	6.1 (2.2)	275 (60)	3.1 (0.6)	20 (10)	1.7 (0.8)
Insurance status								
Medicare	2581 (157)	5.4 (0.3)	976 (78)	3.6 (0.2)	1369 (99)	8.1 (0.5)	308 (46)	4.6 (0.6)
Medicaid	12 127 (923)	25.3 (1.1)	7220 (647)	26.6 (1.5)	3749 (304)	22.2 (1.1)	1598 (194)	24.0 (2.3)
Private	29 064 (1698)	60.5 (1.2)	16 547 (1278)	60.9 (1.6)	10 589 (657)	62.8 (1.1)	3634 (352)	54.5 (2.6)
Self-pay	2814 (285)	5.9 (0.5)	1489 (177)	5.5 (0.5)	747 (125)	4.4 (0.7)	797 (143)	11.9 (1.9)
Other	1097 (204)	2.3 (0.4)	723 (181)	2.7 (0.6)	329 (67)	2.0 (0.4)	280 (110)	4.2 (1.6)
Unknown	337 (107)	0.7 (0.2)	232 (88)	0.9 (0.3)	89 (35)	0.5 (0.2)	53 (23)	0.8 (0.3)
Income status								
Low	9604 (519)	20.0 (0.5)	5547 (370)	20.4 (0.7)	3298 (208)	19.5 (0.7)	1248 (108)	18.7 (1.1)
Medium low	10 520 (635)	21.9 (0.6)	5796 (442)	21.3 (0.8)	4099 (266)	24.3 (0.7)	1236 (106)	18.5 (0.9)
Medium high	12 667 (795)	26.4 (0.5)	7282 (557)	26.8 (0.6)	4482 (317)	26.6 (0.8)	1657 (151)	24.8 (1.1)
High	14 325 (985)	29.8 (1.0)	8220 (748)	30.2 (1.3)	4636 (338)	27.5 (1.0)	2305 (241)	34.6 (1.6)
Unknown	904 (96)	1.9 (0.2)	342 (45)	1.3 (0.1)	357 (51)	2.1 (0.3)	224 (48)	3.4 (0.6)
Hospital location or teaching status								
Rural	480 (132)	1.0 (0.3)	334 (126)	1.2 (0.5)	148 (20)	0.9 (0.1)	1 (1)	0
Urban nonteaching	5072 (585)	10.6 (1.2)	2302 (350)	8.5 (1.3)	2430 (399)	14.4 (2.2)	699 (124)	10.5 (1.9)
Urban teaching	42 467 (2630)	88.4 (1.3)	24 551 (1907)	90.3 (1.4)	14 293 (931)	84.7 (2.2)	5970 (528)	89.5 (1.9)
Hospital bed size, inpatient^b^								
Small	3620 (694)	34.1 (4.8)	255 (57)	25.9 (5.1)	3270 (611)	36.6 (5.0)	345 (125)	28.9 (8.7)
Medium	2015 (356)	19.0 (3.1)	145 (44)	14.7 (4.2)	1425 (285)	16.0 (3.0)	490 (165)	41.0 (9.7)
Large	4990 (535)	47.0 (4.4)	585 (93)	59.4 (5.8)	4230 (515)	47.4 (4.7)	360 (88)	30.1 (7.3)
Hospital bed size, hospital ambulatory surgery^b^								
Small	1749 (331)	4.7 (0.9)	1176 (247)	4.5 (1.0)	373 (66)	4.7 (0.9)	259 (94)	4.7 (1.7)
Medium	12 041 (1540)	32.2 (3.3)	8592 (1293)	32.8 (3.8)	2139 (208)	26.9 (2.6)	2145 (369)	39.2 (4.7)
Large	23 604 (1980)	63.1 (3.3)	16 433 (1426)	62.7 (3.8)	5435 (508)	68.4 (2.8)	3069 (316)	56.1 (4.7)
Hospital region								
Northeast	12 396 (1189)	25.8 (2.3)	7054 (817)	25.9 (2.8)	4695 (548)	27.8 (2.7)	1208 (187)	18.1 (2.7)
Midwest	6881 (607)	14.3 (1.3)	4198 (464)	15.4 (1.8)	2514 (227)	14.9 (1.4)	826 (157)	12.4 (2.3)
South	6705 (688)	14.0 (1.4)	3572 (494)	13.1 (1.8)	2597 (274)	15.4 (1.6)	864 (132)	13.0 (2.0)
West	22 037 (2242)	45.9 (2.9)	12 362 (1627)	45.5 (3.7)	7065 (774)	41.9 (3.1)	3772 (466)	56.6 (3.8)
HIV or AIDS	421 (51)	0.9 (0.1)	204 (32)	0.7 (0.1)	125 (23)	0.7 (0.1)	110 (21)	1.6 (0.3)
Substance abuse	158 (27)	0.3 (0.1)	66 (15)	0.2 (0.1)	78 (19)	0.5 (0.1)	22 (8)	0.3 (0.1)
Alcohol abuse	158 (27)	0.3 (0.1)	66 (15)	0.2 (0.1)	78 (19)	0.5 (0.1)	22 (8)	0.3 (0.1)
Drug abuse	0	0	0	0	0	0	0	0
Mental health	7351 (419)	15.3 (0.7)	4077 (315)	15.0 (0.9)	2693 (168)	16.0 (0.8)	1072 (118)	16.1 (1.1)
Psychoses	186 (23)	0.4 ( 0)	84 (11)	0.3 ( 0)	73 (15)	0.4 (0.1)	42 (12)	0.6 (0.2)
Depression	7192 (412)	15.0 (0.7)	4012 (311)	14.8 (0.9)	2631 (165)	15.6 (0.8)	1034 (116)	15.5 (1.1)

^a^ Race was only available in inpatient encounters (National Inpatient Sample).

^b^ Different cutoff was used to define bed size in inpatient encounters (National Inpatient Sample) and hospital ambulatory surgery encounters (Nationwide Ambulatory Surgery Sample).

**Figure 1.**
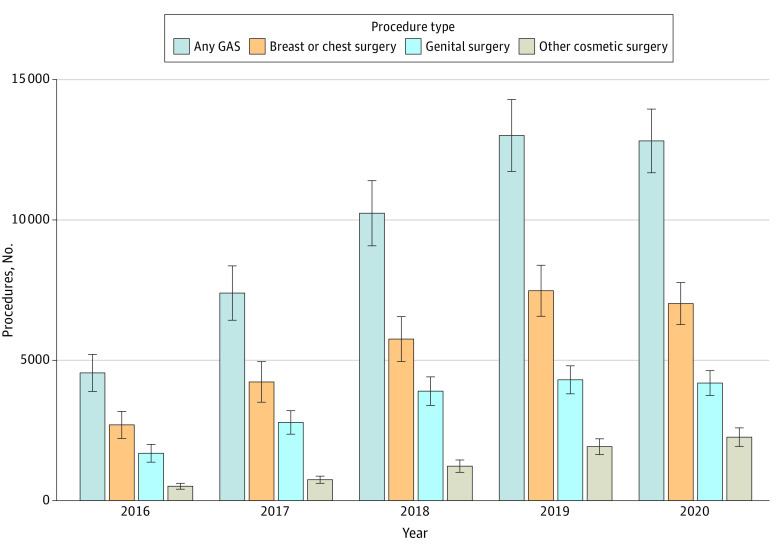
Gender-Affirming Surgical Procedures Performed by Year Stratified by Type Error bars represent 95% CIs. GAS indicates gender-affirming surgery.

The overall number of health system encounters for gender identity disorder rose from 13 855 in 2016 to 38 470 in 2020. Among encounters with a billing code for gender identity disorder, there was a consistent rise in the percentage that were for GAS from 4552 (32.9%) in 2016 to 13 011 (37.1%) in 2019, followed by a decline to 12 818 (33.3%) in 2020 (Figure 1 and eFigure in Supplement 1). Among patients undergoing ambulatory surgical procedures, 37 394 (80.3%) of the surgical procedures included gender-affirming surgical procedures. For those with hospital admissions with gender identity disorder, 10 625 (11.8%) of admissions were for GAS.

Breast and chest procedures were most common and were performed for 27 187 patients (56.6%). Genital reconstruction was performed for 16 872 patients (35.1%), and other facial and cosmetic procedures for 6669 patients (13.9%) (Table 2). The most common individual procedure was breast reconstruction in 21 244 (44.2%), while the most common genital reconstructive procedure was hysterectomy (4489 [9.3%]), followed by orchiectomy (3425 [7.1%]), and vaginoplasty (3381 [7.0%]). Among patients who underwent other facial and cosmetic procedures, liposuction (2945 [6.1%]) was most common, followed by rhinoplasty (2446 [5.1%]) and facial feminizing surgery and chin augmentation (1874 [3.9%]).

**Table 2.** Number of Gender-Affirming Procedures Overall

Surgical procedure	No. (SE)	% (SE)
Gender-affirming surgery	48 019 (2697)	NA
Breast or chest surgery	27 187 (1942)	56.6 (1.7)
Breast reconstruction	21 244 (1646)	44.2 (1.7)
Mammaplasty	4926 (375)	10.3 (0.5)
Mastopexy or nipple reconstruction	10 234 (1009)	21.3 (1.3)
Genital surgery	16 872 (1013)	35.1 (1.6)
Orchitectomy	3425 (288)	7.1 (0.5)
Prostatectomy	22 (9)	0
Penectomy	671 (122)	1.4 (0.3)
Vaginoplasty	3381 (427)	7.0 (0.9)
Clitoroplasty or labiaplasty	424 (62)	0.9 (0.1)
Hysterectomy	4489 (229)	9.3 (0.5)
Salpingo-oophorectomy	666 (57)	1.4 (0.1)
Vaginectomy	272 (68)	0.6 (0.1)
Vulvectomy	39 (11)	0.1 (0)
Metoidioplasty or phalloplasty	1226 (265)	2.6 (0.5)
Urethroplasty	2233 (277)	4.6 (0.6)
Scrotoplasty	217 (39)	0.5 (0.1)
Testicular prostheses	400 (82)	0.8 (0.2)
GAS NOS	3760 (464)	7.8 (1.0)
Other cosmetic procedures	6669 (542)	13.9 (0.9)
Rhinoplasty	2446 (315)	5.1 (0.6)
Rhytidectomy	1721 (257)	3.6 (0.5)
Blepharoplasty	219 (36)	0.5 (0.1)
Hair removal or hair transplantation	10 (7)	0
Facial feminizing or chin augmentation	1874 (257)	3.9 (0.5)
Liposuction	2945 (270)	6.1 (0.5)
Collagen injections	64 (21)	0.1 (0)
Trachea shave or reduction thyroid chondroplasty	632 (101)	1.3 (0.2)
Other	447 (82)	0.9 (0.2)
No. of surgical groups		
1	45 333 (2573)	94.4 (0.4)
2	2664 (243)	5.5 (0.4)
3	22 (8)	0
No. of individual procedures		
1	31 668 (1739)	65.9 (1.3)
2	13 415 (1075)	27.9 (1.2)
3	2338 (219)	4.9 (0.4)
4	532 (72)	1.1 (0.1)
5	56 (20)	0.1 (0)
6	11 (7)	0
Mean (SE)	1.42 (0.02)	NA

Abbreviations: GAS, gender-affirming surgery; NA, not available; NOS, not otherwise specified.

The absolute number of GAS procedures rose from 4552 in 2016 to a peak of 13 011 in 2019 and then declined slightly to 12 818 in 2020 (Figure 1). Similar trends were noted for breast and chest surgical procedures as well as genital surgery, while the rate of other facial and cosmetic procedures increased consistently from 2016 to 2020. The distribution of the individual procedures performed in each class were largely similar across the years of analysis (Table 3).

**Table 3.** Number of GAS Procedures by Year

Characteristics	2016		2017		2018		2019		2020	
	No. (SE)	% (SE)	No. (SE)	% (SE)	No. (SE)	% (SE)	No. (SE)	% (SE)	No. (SE)	% (SE)
GAS	4552 (658)	9.5 (1.4)	7397 (968)	15.4 (1.6)	10 242 (1162)	21.3 (1.8)	13 011 (1280)	27.1 (2.4)	12 818 (1136)	26.7 (2.2)
Breast or chest surgery	2700 (483)	9.9 (1.8)	4229 (723)	15.6 (2.0)	5757 (799)	21.2 (2.1)	7479 (907)	27.5 (3.0)	7022 (747)	25.8 (2.7)
Breast reconstruction	2027 (404)	9.5 (1.9)	3319 (618)	15.6 (2.2)	4582 (687)	21.6 (2.3)	6090 (781)	28.7 (3.3)	5226 (586)	24.6 (2.7)
Mammaplasty	577 (117)	11.7 (2.3)	788 (141)	16.0 (2.2)	1056 (160)	21.4 (2.4)	1272 (172)	25.8 (3.1)	1233 (143)	25.0 (2.8)
Mastopexy or nipple reconstruction	1014 (256)	9.9 (2.5)	1582 (399)	15.5 (3.0)	2120 (394)	20.7 (2.8)	2939 (519)	28.7 (4.4)	2580 (347)	25.2 (3.5)
Genital surgery	1689 (317)	10.0 (1.8)	2787 (418)	16.5 (2.2)	3901 (509)	23.1 (2.5)	4305 (500)	25.5 (2.6)	4190 (439)	24.8 (2.4)
Orchitectomy	394 (87)	11.5 (2.4)	514 (90)	15.0 (2.2)	732 (140)	21.4 (3.2)	830 (119)	24.2 (3.2)	955 (147)	27.9 (3.7)
Prostatectomy	5 (5)	22.7 (19.3)	0	0	5 (5)	22.7 (19.3)	4 (2)	19.0 (11.8)	8 (5)	35.6 (19.9)
Penectomy	75 (36)	11.2 (5.1)	66 (22)	9.9 (3.4)	86 (32)	12.8 (4.7)	162 (41)	24.2 (6.2)	281 (102)	41.9 (9.8)
Vaginoplasty	310 (114)	9.2 (3.3)	541 (212)	16.0 (5.6)	790 (248)	23.4 (6.2)	831 (194)	24.6 (5.2)	908 (188)	26.9 (5.1)
Clitoroplasty or labiaplasty	35 (13)	8.2 (3.1)	55 (20)	13.0 (4.1)	78 (27)	18.5 (5.3)	111 (27)	26.0 (5.8)	146 (37)	34.4 (7.0)
Hysterectomy	461 (52)	10.3 (1.2)	837 (85)	18.6 (1.4)	1059 (105)	23.6 (1.7)	971 (93)	21.6 (1.9)	1160 (106)	25.8 (2.1)
Salpingo-oophorectomy	99 (22)	14.8 (3.0)	146 (34)	22.0 (4.3)	133 (23)	20.0 (3.2)	139 (24)	20.8 (3.3)	149 (22)	22.4 (3.2)
Vaginectomy	69 (51)	25.3 (14.5)	39 (15)	14.2 (5.8)	54 (20)	19.8 (7.5)	27 (13)	9.9 (4.8)	84 (36)	30.7 (11.2)
Vulvectomy	3 (2)	8.0 (5.7)	3 (3)	7.6 (7.3)	4 (3)	11.1 (8.4)	10 (6)	25.5 (13.4)	19 (8)	47.8 (14.5)
Metoidioplasty or phalloplasty	224 (126)	18.3 (9.1)	261 (133)	21.3 (9.4)	236 (134)	19.2 (9.5)	284 (117)	23.1 (8.6)	222 (77)	18.1 (6.4)
Urethroplasty	119 (38)	5.3 (1.7)	346 (108)	15.5 (4.5)	567 (172)	25.4 (6.3)	624 (140)	27.9 (5.5)	577 (124)	25.8 (5.0)
Scrotoplasty	21 (11)	9.8 (4.9)	31 (13)	14.2 (4.9)	49 (18)	22.6 (6.3)	62 (17)	28.7 (7.3)	54 (16)	24.8 (6.8)
Testicular prostheses	48 (30)	12.0 (7.0)	54 (27)	13.4 (5.6)	79 (35)	19.6 (7.0)	108 (36)	27.1 (8.3)	112 (38)	27.9 (8.6)
GAS NOS	275 (148)	7.3 (3.7)	535 (180)	14.2 (4.4)	925 (228)	24.6 (5.3)	1155 (262)	30.7 (5.8)	870 (205)	23.1 (4.9)
Other cosmetic procedures	513 (105)	7.7 (1.6)	745 (129)	11.2 (1.7)	1228 (220)	18.4 (2.8)	1922 (280)	28.8 (3.6)	2262 (329)	33.9 (3.9)
Rhinoplasty	99 (30)	4.0 (1.3)	237 (69)	9.7 (2.7)	408 (120)	16.7 (4.4)	761 (161)	31.1 (5.7)	942 (220)	38.5 (6.6)
Rhytidectomy	72 (28)	4.2 (1.7)	204 (74)	11.9 (4.0)	295 (111)	17.1 (5.7)	521 (126)	30.3 (6.5)	629 (173)	36.6 (7.6)
Blepharoplasty	17 (7)	7.6 (3.1)	47 (15)	21.3 (5.6)	49 (22)	22.5 (7.9)	72 (16)	33.1 (6.9)	34 (10)	15.5 (4.5)
Hair removal or hair transplantation	5 (5)	50.0 (35.4)	0	0	5 (5)	50.0 (35.4)	0	0	0	0
Facial feminizing or chin augmentation	68 (25)	3.7 (1.4)	152 (52)	8.1 (2.6)	298 (104)	15.9 (5.0)	577 (123)	30.8 (5.9)	779 (186)	41.5 (7.0)
Liposuction	348 (85)	11.8 (2.8)	397 (78)	13.5 (2.1)	655 (139)	22.2 (3.5)	773 (120)	26.2 (3.7)	773 (104)	26.2 (3.4)
Collagen injections	4 (2)	6.2 (3.9)	17 (11)	26.5 (10.6)	21 (10)	33.4 (8.2)	10 (4)	15.2 (7.2)	12 (5)	18.7 (8.3)
Trachea shave or reduction thyroid chondroplasty	22 (9)	3.5 (1.5)	58 (19)	9.2 (2.9)	72 (23)	11.4 (3.5)	203 (54)	32.1 (7.3)	276 (74)	43.7 (8.1)
Other	4 (2)	0.9 (0.5)	14 (5)	3.0 (1.2)	29 (14)	6.5 (3.2)	24 (15)	5.4 (3.4)	376 (78)	84.1 (5.2)

Abbreviations: GAS, gender-affirming surgery; NOS, not otherwise specified.

**Figure 2.**
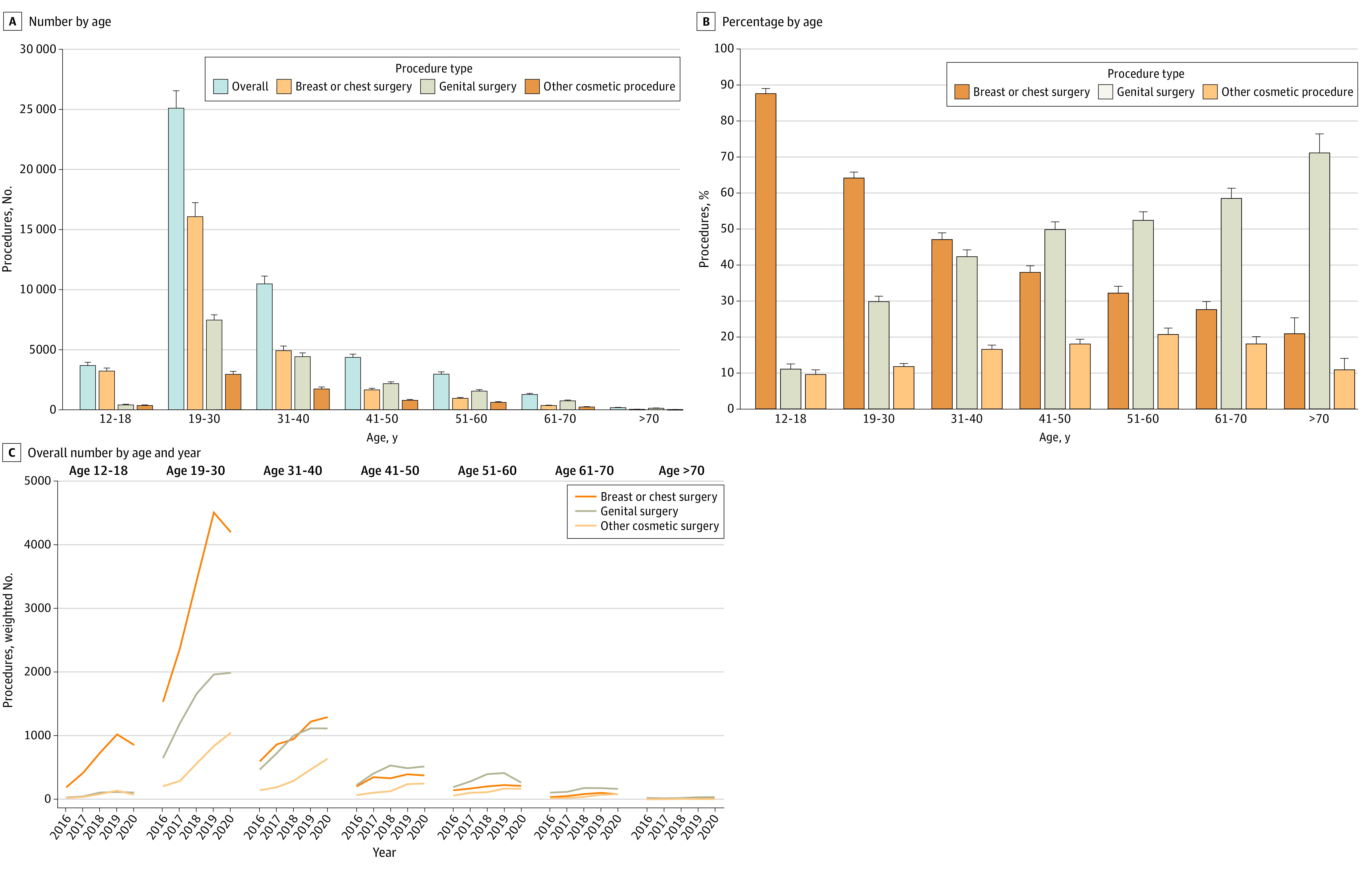
Gender-Affirming Surgical Procedures Performed During the Study Stratified by Age Percentages are based on the number of procedures divided by number of patients; thus, as some patients underwent multiple procedures the total may be greater than 100%. Error bars represent 95% CIs.

When stratified by age, patients 19 to 30 years had the greatest number of procedures, 25 099 (Figure 2). There were 10 476 procedures performed in those aged 31 to 40 years and 4359 in those aged 41 to 50 years. Among patients younger than 19 years, 3678 GAS procedures were performed. GAS was less common in those cohorts older than 50 years. Overall, the greatest number of breast and chest surgical procedures, genital surgical procedures, and facial and other cosmetic surgical procedures were performed in patients aged 19 to 30 years.

When stratified by the type of procedure performed, breast and chest procedures made up the greatest percentage of the surgical interventions in younger patients while genital surgical procedures were greater in older patients (Figure 2). Additionally, 3215 patients (87.4%) aged 12 to 18 years underwent GAS and had breast or chest procedures. This decreased to 16 067 patients (64.0%) in those aged 19 to 30 years, 4918 (46.9%) in those aged 31 to 40 years, and 1650 (37.9%) in patients aged 41 to 50 years (*P* < .001). In contrast, 405 patients (11.0%) aged 12 to 18 years underwent genital surgery. The percentage of patients who underwent genital surgery rose sequentially to 4423 (42.2%) in those aged 31 to 40 years, 1546 (52.3%) in those aged 51 to 60 years, and 742 (58.4%) in those aged 61 to 70 years (*P* < .001). The percentage of patients who underwent facial and other cosmetic surgical procedures rose with age from 9.5% in those aged 12 to 18 years to 20.6% in those aged 51 to 60 years, then gradually declined (*P* < .001). Figure 2 displays the absolute number of procedure classes performed by year stratified by age. The greatest magnitude of the decline in 2020 was in younger patients and for breast and chest procedures.

## Discussion

These findings suggest that the number of GAS procedures performed in the US has increased dramatically, nearly tripling from 2016 to 2019. Breast and chest surgery is the most common class of procedure performed while patients are most likely to undergo surgery between the ages of 19 and 30 years. The number of genital surgical procedures performed increased with increasing age.

Consistent with prior studies, we identified a remarkable increase in the number of GAS procedures performed over time.^9,16^ A prior study examining national estimates of inpatient GAS procedures noted that the absolute number of procedures performed nearly doubled between 2000 to 2005 and from 2006 to 2011. In our analysis, the number of GAS procedures nearly tripled from 2016 to 2020.^9,17^ Not unexpectedly, a large number of the procedures we captured were performed in the ambulatory setting, highlighting the need to capture both inpatient and outpatient procedures when analyzing data on trends. Like many prior studies, we noted a decrease in the number of procedures performed in 2020, likely reflective of the COVID-19 pandemic.^18^ However, the decline in the number of procedures performed between 2019 and 2020 was relatively modest, particularly as these procedures are largely elective.

Analysis of procedure-specific trends by age revealed a number of important findings. First, GAS procedures were most common in patients aged 19 to 30 years. This is in line with prior work that demonstrated that most patients first experience gender dysphoria at a young age, with approximately three-quarters of patients reporting gender dysphoria by age 7 years. These patients subsequently lived for a mean of 23 years for transgender men and 27 years for transgender women before beginning gender transition treatments.^19^ Our findings were also notable that GAS procedures were relatively uncommon in patients aged 18 years or younger. In our cohort, fewer than 1200 patients in this age group underwent GAS, even in the highest volume years. GAS in adolescents has been the focus of intense debate and led to legislative initiatives to limit access to these procedures in adolescents in several states.^20,21^

Second, there was a marked difference in the distribution of procedures in the different age groups. Breast and chest procedures were more common in younger patients, while genital surgery was more frequent in older individuals. In our cohort of individuals aged 19 to 30 years, breast and chest procedures were twice as common as genital procedures. Genital surgery gradually increased with advancing age, and these procedures became the most common in patients older than 40 years. A prior study of patients with commercial insurance who underwent GAS noted that the mean age for mastectomy was 28 years, significantly lower than for hysterectomy at age 31 years, vaginoplasty at age 40 years, and orchiectomy at age 37 years.^16^ These trends likely reflect the increased complexity of genital surgery compared with breast and chest surgery as well as the definitive nature of removal of the reproductive organs.

### Limitations

This study has limitations. First, there may be under-capture of both transgender individuals and GAS procedures. In both data sets analyzed, gender is based on self-report. NIS specifically makes notation of procedures that are considered inconsistent with a patient’s reported gender (eg, a male patient who underwent oophorectomy). Similar to prior work, we assumed that patients with a code for gender identity disorder or transsexualism along with a surgical procedure classified as inconsistent underwent GAS.^9^ Second, we captured procedures commonly reported as GAS procedures; however, it is possible that some of these procedures were performed for other underlying indications or diseases rather than solely for gender affirmation. Third, our trends showed a significant increase in procedures through 2019, with a decline in 2020. The decline in services in 2020 is likely related to COVID-19 service alterations. Additionally, while we comprehensively captured inpatient and ambulatory surgical procedures in large, nationwide data sets, undoubtedly, a small number of procedures were performed in other settings; thus, our estimates may underrepresent the actual number of procedures performed each year in the US.

## Conclusions

These data have important implications in providing an understanding of the use of services that can help inform care for transgender populations. The rapid rise in the performance of GAS suggests that there will be a greater need for clinicians knowledgeable in the care of transgender individuals and with the requisite expertise to perform GAS procedures. However, numerous reports have described the political considerations and challenges in the delivery of transgender care.^22^ Despite many medical societies recognizing the necessity of gender-affirming care, several states have enacted legislation or policies that restrict gender-affirming care and services, particularly in adolescence.^20,21^ These regulations are barriers for patients who seek gender-affirming care and provide legal and ethical challenges for clinicians. As the use of GAS increases, delivering equitable gender-affirming care in this complex landscape will remain a public health challenge.

## Notes

Supplement 1. **eTable.** *ICD-10* and CPT Codes of Gender-Affirming Surgery **eFigure. **Percentage of Patients With Codes for Gender Identity Disorder Who Underwent GAS

Supplement 2.
Data Sharing Statement
