# Supplementary material for: National Estimates of Gender-Affirming Surgery in the US
Source: JAMA Netw Open. 2023 Aug 23;6(8):e2330348. doi: 10.1001/jamanetworkopen.2023.30348 (PMC10448302; doi:10.1001/jamanetworkopen.2023.30348)
Supplement: Supplement 1. — eTable. ICD-10 and CPT Codes of Gender-Affirming Surgery eFigure. Percentage of Patients With Codes for Gender Identity Disorder Who Underwent GAS [file jamanetwopen-e2330348-s001.pdf]

Supplemental Online Content

Wright JD, Chen L, Suzuki Y, Matsuo K, Hershman DL. National estimates of gender affirming surgery in the US. *JAMA Netw Open*. 2023;6(8):e2330348. doi:10.1001/jamanetworkopen.2023.30348

eTable. *ICD-10* and *CPT* Codes of Gender-Affirming Surgery

eFigure. eFigure. Percentage of Patients With Codes for Gender Identity Disorder Who Underwent GAS

This supplemental material has been provided by the authors to give readers additional information about their work.

© 2023 Wright JD et al. *JAMA Network Open*

eTable. *ICD-10* and *CPT* Codes of Gender-Affirming Surgery

	CPT	ICD-10
**Breast or chest surgery**		
Breast reconstruction	19303, 19304;	0HTV0ZZ;
Mammaplasty	19325;	0H0V*, 0HRV*, 0HHV0NZ, 0HHV3NZ, 0HHV7NZ, 0HHV8NZ;
Mastopexy or nipple reconstruction	19316, 19350;	0HQV*, 0HSV0*, 0HSW*, 0HSX*;
**Genital surgery**		
Orchitectomy	54520, 54690;	0VTC*;
Prostatectomy	55840, 55866;	0VT0*;
Penectomy	54125;	0VTS*;
Vaginoplasty	57291, 57292, 57295, 57296, 57335, 57426;	0W4M*;
Clitoroplasty or labiaplasty	55970, 56800, 56805;	0W0M*;
Hysterectomy	58150, 58152, 58180, 58200, 58950, 58951, 58956, 58260, 58262, 58263, 58267, 58270, 58275, 58280, 58290, 58291, 58292, 58293, 58294, 58541, 58542, 58543, 58544, 58550, 58552, 58553, 58554, 58570, 58571, 58572, 58573;	0UT90*; 0UT97*; 0UT94*, 0UT98*, 0UT9F *;
Salpingo-oophorectomy	58720, 58940, 58943, 58661;	0UT2*, 0UT7*;
Vaginectomy	57106, 57110;	0UTG*;
Vulvectomy	56625;	0UTM*;
Metoidioplasty or phalloplasty	55899, 55980;	0W4N*;
Urethroplasty	53420, 53425, 53430;	0TQD*, 0TRD*, 0TUD*;
Scrotoplasty	55175, 55180;	
Testicular prostheses	54660;	0VRC0JZ;
**Other cosmetic procedures**		
Rhinoplasty	30400, 30410, 30420, 30430, 30435, 30450;	090K*, 09RK*, 09UK*;
Rhytidectomy	15824, 15825, 15826, 15828, 15829;	0J01*, 0W02*, 0WU2*;
Blepharoplasty	15820, 15821, 15822, 15823;	080N*, 080P*, 080Q*, 080R*;
Hair removal or hair transplantation	15775, 15776, 17380;	0HRS*;
Facial feminizing or chin augmentation	21120, 21121, 21122, 21123, 21125, 21127, 21208, 21209;	0NRT*, 0NRV*, 0NUT*, 0NUV*, 0W04*, 0W05*;
Liposuction	15830, 15832, 15833, 15834, 15835, 15836, 15837, 15838, 15839, 15876, 15877, 15878, 15879;	
Collagen injections	11950, 11951, 11952, 11954;	

© 2023 Wright JD et al. *JAMA Network Open*

Trachea shave or reduction, thyroid chondroplasty	31599, 31899;	0BQ1*, 0CRS*, 0CUS*;
Other	14021, 14060, 15769, 15770, 15771, 15772, 15773, 15774, 15775;	

© 2023 Wright JD et al. *JAMA Network Open*

eFigure. Percentage of Patients With Codes for Gender Identity Disorder Who Underwent GAS

Shown overall, for inpatient admissions and for ambulatory surgery encounters. Error bars represent 95% confidence intervals.

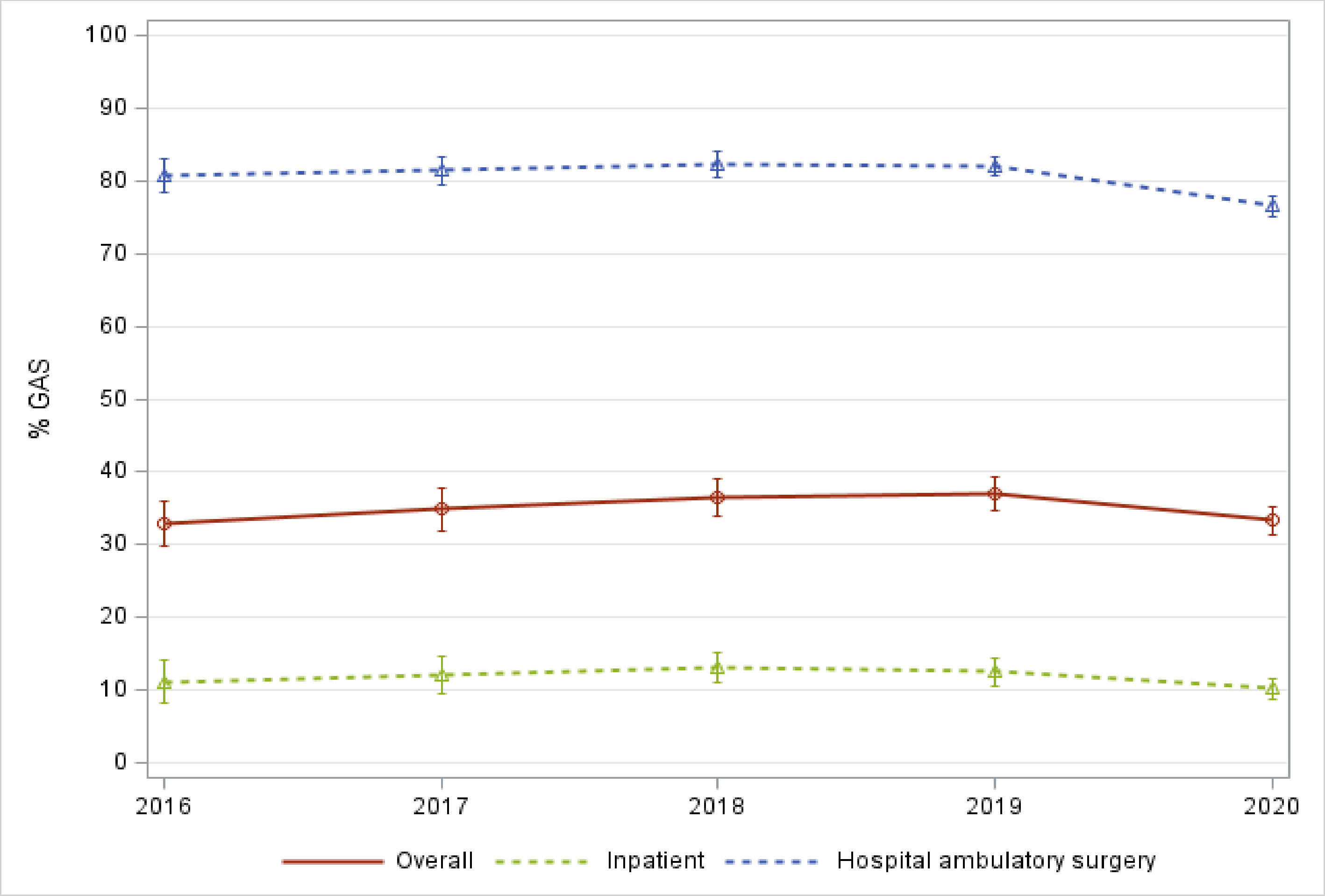

© 2023 Wright JD et al. *JAMA Network Open*
