# Supplementary material for: National Estimates of Gender-Affirming Surgery in the US
Source: JAMA Netw Open. 2023 Aug 23;6(8):e2330348. doi: 10.1001/jamanetworkopen.2023.30348 (PMC10448302; doi:10.1001/jamanetworkopen.2023.30348)
Supplement: Supplement 2. — Data Sharing Statement [file jamanetwopen-e2330348-s002.pdf]

Data Sharing Statement

Wright. National Estimates of Gender-Affirming Surgery in the US. *JAMA Netw Open*.
Published August 23, 2023. doi:10.1001/jamanetworkopen.2023.30348

Data
Data available: No

Additional Information
Explanation for why data not available: Data publicly available for investigators
